# Sequencing vs. amplification for the estimation of allele dosages in sugarcane (*Saccharum* spp.)

**DOI:** 10.1002/aps3.11574

**Published:** 2024-03-13

**Authors:** Hugo Jaimes, Alejandra Londoño, Carolina Saavedra‐Diaz, Jhon Henry Trujillo‐Montenegro, Jershon López‐Gerena, John J. Riascos, Fernando S. Aguilar

**Affiliations:** ^1^ Colombian Sugarcane Research Center Cenicaña Calle 38N 3CN‐75 Cali Valle del Cauca Colombia; ^2^ Pontificia Universidad Javeriana Calle 18 118–250 Cali Colombia

**Keywords:** Flex‐Seq, genotyping‐by‐sequencing (GBS), kompetitive allele‐specific PCR (KASP), restriction site–associated DNA sequencing (RADseq), sugarcane

## Abstract

**Premise:**

Detecting single‐nucleotide polymorphisms (SNPs) in a cost‐effective way is fundamental in any plant breeding pipeline. Here, we compare three genotyping techniques for their ability to reproduce the allele dosage of SNPs of interest in sugarcane (*Saccharum* spp.).

**Methods:**

To identify a reproducible technique to estimate allele dosage for the validation of SNP markers, the correlation between Flex‐Seq, kompetitive allele‐specific PCR (KASP), and genotyping‐by‐sequencing and restriction site–associated DNA sequencing (GBS+RADseq) was determined for a set of 76 SNPs. To find alternative methodologies for allele dosage estimation, the KASP and Flex‐Seq techniques were compared for the same set of SNPs. For the three techniques, a population of 53 genotypes from the diverse sugarcane panel of the Centro de Investigación de la Caña de Azúcar (Cenicaña), Colombia, was selected.

**Results:**

The average Pearson correlation coefficients between GBS+RADseq and Flex‐Seq, GBS+RADseq and KASP, and Flex‐Seq and KASP were 0.62 ± 0.27, 0.38 ± 0.27, and 0.38 ± 0.30, respectively.

**Discussion:**

Flex‐Seq reproduced the allele dosages determined using GBS+RADseq with good levels of precision because of its depth of sequencing and ability to target specific positions in the genome. Additionally, Flex‐Seq outperformed KASP by allowing the conversion of a higher number of SNPs and a more accurate estimation of the allele dosage. Flex‐Seq has therefore become the genotyping methodology of choice for marker validation at Cenicaña.

## Resumen

**Premisa:**

Detectar polimorfismos de un único nucleótido (SNP) de forma costo‐efectiva es fundamental en cualquier programa de mejoramiento genético. En este artículo nosotros comparamos tres técnicas de genotipado para medir su habilidad en reproducir las dosis alélicas de SNPs de interés en caña de azúcar (*Saccharum* spp.).

**Métodos:**

Para identificar una técnica reproducible para la estimación de dosis alélicas durante los pasos de validación de marcadores, la correlación entre Flex‐Seq, kompetitive allele‐specific PCR (KASP), y genotyping‐by‐sequencing and restriction site–associated DNA sequencing (GBS+RADseq) fue determinada para un set de 76 SNPs. Para identificar metodologías alternativas en la estimación de las dosis alélicas, las tecnologías KASP y Flex‐Seq fueron comparadas para el mismo grupo de SNPs. Para las tres técnicas, una población de 53 genotipos fue seleccionados de la población diversa de caña de azúcar del Centro de Investigación de la Caña de Azúcar (Cenicaña), Colombia.

**Resultados:**

El promedio del coeficiente de correlación de Pearson entre GBS+RADseq y Flex‐Seq, GBS+RADseq y KASP, y Flex‐Seq y KASP fue de 0.62 ± 0.27, 0.38 ± 0.27, y 0.38 ± 0.30, respectivamente.

**Discusión:**

Flex‐Seq reprodujo las dosis alélicas determinadas usando GBS+RADseq con buenos niveles de precisión debido a su profundidad de secuenciación y habilidad de secuenciar posiciones especificas en el genoma. Adicionalmente, Flex‐Seq superó a KASP al permitir la conversión de un número mayor de SNPs y al estimar las dosis alélicas de forma más precisa. Flex‐Seq por tanto se convierte en la metodología de genotipado de elección para la validación de marcadores en Cenicaña.

Sugarcane (*Saccharum* L. spp.) is a highly polyploid and aneuploid organism, derived from the interspecific cross between *Saccharum officinarum* L. and *S. spontaneum* L., two ancestral species with different chromosome numbers (Gouy et al., 2014). *Saccharum officinarum* (2*n* = 80) contributed almost 80% of the genome of modern cultivars, while *S. spontaneum* (2*n* = 40–128) material represents almost 10% of the genome (Garsmeur et al., 2018; Sharma et al., 2018; Piperidis and D'Hont, 2020). The remaining 10% is believed to comprise recombinant chromosomes containing genetic material from both ancestral species (Heller‐Uszynska et al., 2010; Racedo et al., 2016). This genetic complexity of sugarcane imposes a major challenge for the discovery, validation, and application of single‐nucleotide polymorphism (SNP) markers, which are essential for modern breeding programs. Developing and standardizing a rapid and cost‐effective technique for the routine identification and use of SNP markers in sugarcane is therefore essential for improving this crop.

The benefits of molecular markers such as SNPs are commonly implemented with modern sequencing techniques, particularly next‐generation sequencing (NGS), which represented a breakthrough in the genotyping process by making whole‐genome sequencing financially and practically accessible for the average researcher. Despite the advantages of NGS technologies, it is necessary to reduce the complexity of large genomes to ensure accurate SNP calling (Hyten et al., 2010). Genotyping‐by‐sequencing (GBS) (Elshire et al., 2011) and restriction site–associated DNA sequencing (RADseq) (Davey et al., 2011) are techniques that offer a representation of the genome using endonucleases with sufficient genome coverage to identify a higher number of polymorphic variants, making them well suited for marker discovery in genetically complex organisms (Wickland et al., 2017). The optimal restriction enzyme choice depends on several factors, the most important of which are the genome size, multiplexing, and the total number of desired markers (Hamblin et al., 2006). Nevertheless, GBS and RADseq present a limited applicability for marker validation due to the difficulty of targeting specific positions within the genome imposed by their restriction enzyme–based enrichment approach (Cronn et al., 2012; Sonah et al., 2022).

Currently, multiple techniques are used for SNP validation, which vary in their chemistry, cost, and high‐throughput capacity (Paris et al., 2003; Giancola et al., 2006). Selecting the appropriate technique for each investigation depends on several factors, such as the sequence length surrounding the SNP, the number of markers and samples that will be genotyped, and the financial resources available (Mammadov et al., 2012). With the advent of high‐throughput targeted sequencing technologies, the validation of markers associated with a phenotype has shifted towards the identification of DNA sequences that could be sequenced in a rapid and inexpensive way, identifying a specific marker within hundreds or thousands of samples. Targeted sequencing requires a knowledge of the positions of the markers and the adjacent regions used for primer and probe construction, which enables researchers to define the region of interest (Cronn et al., 2012). Flex‐Seq Ex‐L (hereafter referred to as Flex‐Seq; LGC Biosearch Technologies, Teddington, United Kingdom) sequencing technology is a high‐throughput genotyping service based on a hybridization enrichment strategy with less breadth of coverage than GBS and RADseq but with a higher depth of sequencing per nucleotide (Cronn et al., 2012; Sonah et al., 2022). This technology takes advantage of the high specificity of the oligonucleotide probes to hydrogen bond to complementary sequences and accurately target SNP positions in the genome with more than 96% reproducibility between samples (Mamanova et al., 2010; Cronn et al., 2012; Sims et al., 2014; Kappelmann‐Fenzl, 2021). Flex‐Seq requires the design of two specific probes, usually between 150 and 250 bp, that target individual regions of the genome by binding to both sides of the SNP. After the probes bind to the template DNA, the targeted region can be amplified and the sequence extracted (Rapid Genomics, 2023).

Alternatively, the PCR‐based technique called kompetitive allele‐specific PCR (hereafter referred to as KASP; LGC Biosearch Technologies) offers high‐quality genotyping for hundreds of SNPs with reduced error rates (Mammadov et al., 2012; He et al., 2014). KASP uses an allele‐specific oligo extension and fluorescence resonance energy transfer (FRET) for signaling, facilitating a high‐throughput analysis (Mammadov et al., 2012). KASP requires two forward primers, each marked with a fluorophore (e.g., FAM or HEX), and one universal reverse primer (He et al., 2014). This methodology offers a medium‐ to high‐throughput capability with options to analyze 96, 384, or 1592 PCR plates in one run (Aitken, 2022). According to Steele et al. (2021), KASP is a codominant technique that has been successfully implemented to detect insertion–deletion loci as well as SNPs, but it has not been able to detect simple‐sequence repeats (SSRs). However, its success is highly dependent on the primer design, the ability to account for non‐target sequences, and the complexity of the genome (Steele et al., 2021). Cenicaña, part of the Optimización Multiescala In‐silico de Cultivos Agrícolas Sostenibles (OMICAS; In‐Silico Driven Multiscale Optimization of Sustainable Agricultural Crops) alliance (Jaramillo‐Botero et al., 2022), has been actively investigating different approaches to estimating allele dosages for molecular markers; thus, in this study we compare two targeted genotyping techniques in the estimation of allele dosages for the validation of SNP markers in sugarcane. First, the ability of Flex‐Seq and KASP to reproduce the allele dosages estimated with the SNP discovery techniques GBS and RADseq was assessed by correlating the allele dosages. After this comparison, Flex‐Seq was correlated with KASP to determine which could be routinely used to estimate the allele dosages of SNP markers in sugarcane varieties within Cenicaña for validation purposes.

## METHODS

### Plant materials and SNP marker selection

**Figure 1:**
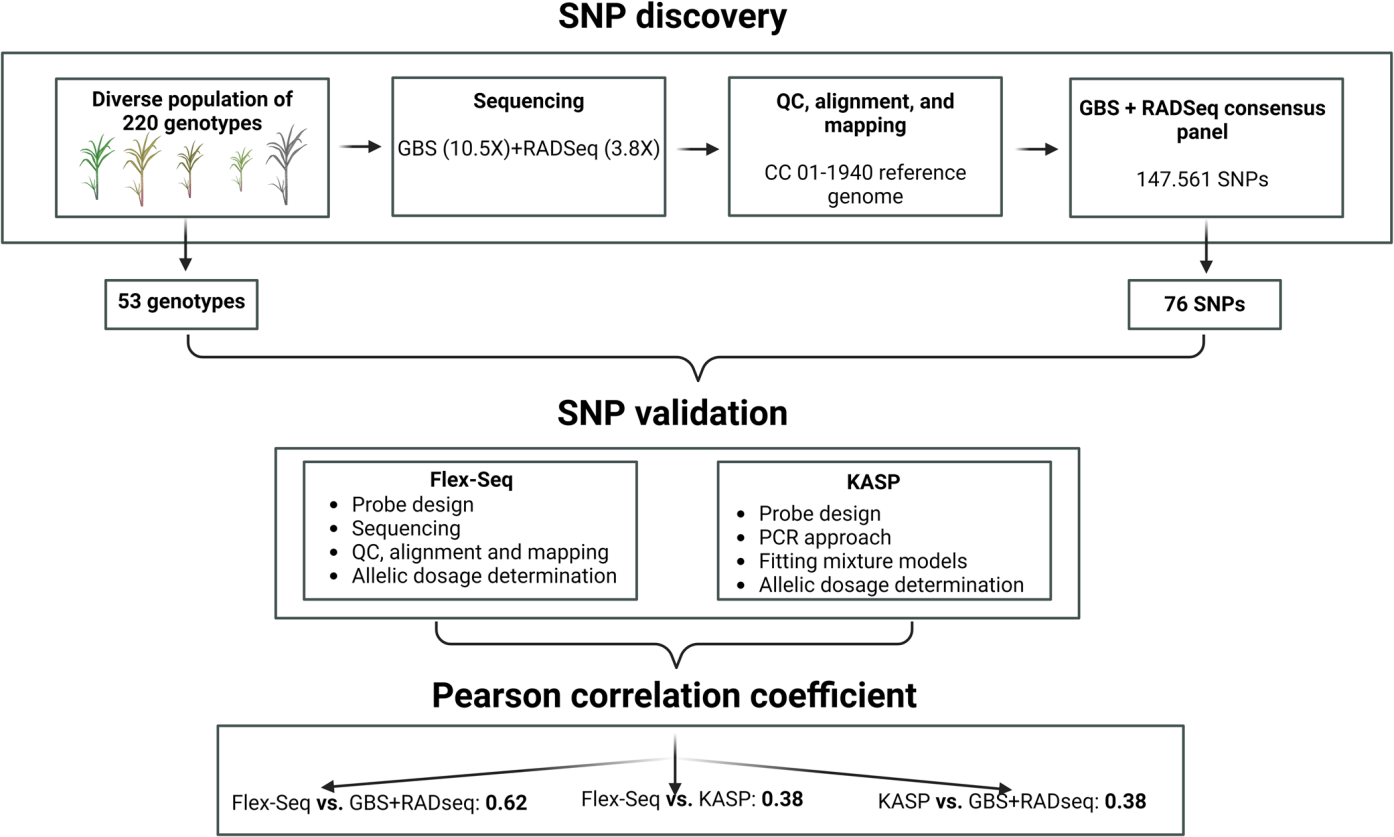
Overview of the experimental workflow as a modular summary. Each module corresponds to a stage of the overall experimental design. QC = quality control. Graphic created with BioRender.com.

A total of 53 sugarcane genotypes were randomly selected from a diverse panel of 220 genotypes. This diverse panel was composed of 98 genotypes that represent the genetic diversity of Cenicaña's germplasm bank (Salazar et al., 2012), 31 wild species, 33 genotypes of relevance for the breeding program (i.e., genetic introductions, commercial varieties, and early selection stage varieties), and 58 genotypes resistant or susceptible to different pests and diseases in Colombia (Appendix S1). The 220 genotypes were previously sequenced through GBS and RADseq for SNP discovery (https://www.ncbi.nlm.nih.gov/bioproject/PRJNA1040257 [accessed 25 January 2024]). Briefly, DNA was extracted by grinding 3 g of midrib‐free leaf tissue in liquid nitrogen, following the protocol of Dellaporta et al. (1983). The DNA was digested with the restriction enzyme *Pst*I for the single‐end GBS technique (Elshire et al., 2011) and with *Eco*RI for the paired‐end RADseq technique (Baird et al., 2008). DNA libraries for both techniques were constructed following the sequencing service providers. Sequencing processes were carried out using an Illumina HiSeq 2000 system (Illumina, San Diego, California, USA) at an average depth of 10.5× for GBS and 3.8× for RADseq. Quality control was determined using FastQC (Andrews, 2010) with default parameters. Cutadapt (Martin, 2011) was used to remove adapters, low‐quality sequences with a Phred score less than 30, and sequences with a length less than 35 bp. Cleaned data from GBS and RADseq were individually aligned and mapped to the CC 01‐1940 reference genome (Trujillo‐Montenegro et al., 2021) using Bowtie version 2.2.5 (Langmead and Salzberg, 2012) with its default parameters. Unique alignments were selected using SAMtools version 1.10 (Li et al., 2009). Both the GBS and RADseq unique alignment data were combined in a consensus panel for variant detection and SNP calling using NGSEP version 4.2.0 (Tello et al., 2022), with a minimum variant quality of 40 (Phred score), a base quality score of 30, a ploidy of 10, a minor allele frequency of 1%, a minimum distance of five nucleotides between SNPs, a calling rate of 50%, and a filter for biallelic SNPs. Finally, a consensus panel, referred to as GBS+RADseq, was produced, with 147,561 SNP markers for each of the 220 genotypes (Figure 1). To evaluate the capability of Flex‐Seq and KASP as reliable baseline methodologies for SNP validation, a subset of 76 polymorphic markers from the GBS+RADseq consensus SNP panel was selected (Figure 1). This subset of markers belongs to a set of SNPs used at Cenicaña to differentiate between genotypes, and to markers associated with agronomic traits of interest.

### KASP and Flex‐Seq primer and probe design

For both KASP and Flex‐Seq, a region of 400 bp (200 bp upstream and 200 bp downstream; Appendix S2) surrounding each SNP was used for primer and probe design (Figure 1). Flex‐Seq includes a multi‐amplification step as part of the enrichment process focused on the region containing the SNP of interest. The probes for this strategy were designed following manufacturer's criteria for the GC content, homopolymer length, off‐target regions, and probe hybridization kinetics. Following probe design, an enrichment PCR‐based step was performed to enable the sequencing process on an Illumina system. For KASP, the company Intertek (Intertek ScanBI Diagnostics, Alnarp, Sweden) designed two specific forward primers, one for each of the alleles (i.e., reference and alternative), and a universal reverse primer (He et al., 2014). Each forward primer has a unique 5′ tail sequence that is complementary to a universal cassette marked with the HEX and FAM fluorophore. KASP sequencing was carried out using Intertek's workflow and genotyping instrumentation.

### Allele dosage estimation and prediction ability

For GBS+RADseq and Flex‐Seq, the allele dosage was estimated by dividing the number of reference alleles (REF) by the sum of the alternative and reference (ALT+REF) alleles $\left(\frac{\mathrm{REF}}{\mathrm{ALT}+\mathrm{REF}}\right)$. The proportions were divided into 11 categories (i.e., 0.0, 0.1, 0.2, 0.3, 0.4, 0.5, 0.6, 0.7, 0.8, 0.9, and 1.0), which represent the possible allelic doses you could find in a decaploid organism such as sugarcane. On the other hand, KASP enables the biallelic scoring of the SNP considering the fluorescence intensity signal from both alleles. In this case, the R (R Core Team, 2022) package fitPoly (Voorrips et al., 2011) was used to calculate the allele dosage by fitting mixture models to the distribution of the fluorescence intensity ratios, assuming a decaploid genotype assignment.

The reproducibility of the Flex‐Seq and KASP findings was assessed by correlating the allele dosages of the 76 SNP markers from GBS+RADseq and those from Flex‐Seq and KASP through a Pearson correlation coefficient (Figure 1). Similarly, the prediction abilities of KASP and Flex‐Seq were compared using the Pearson correlation coefficient to analyze the allele dosages estimated for the same selected markers (Figure 1).

## RESULTS

**Figure 2:**
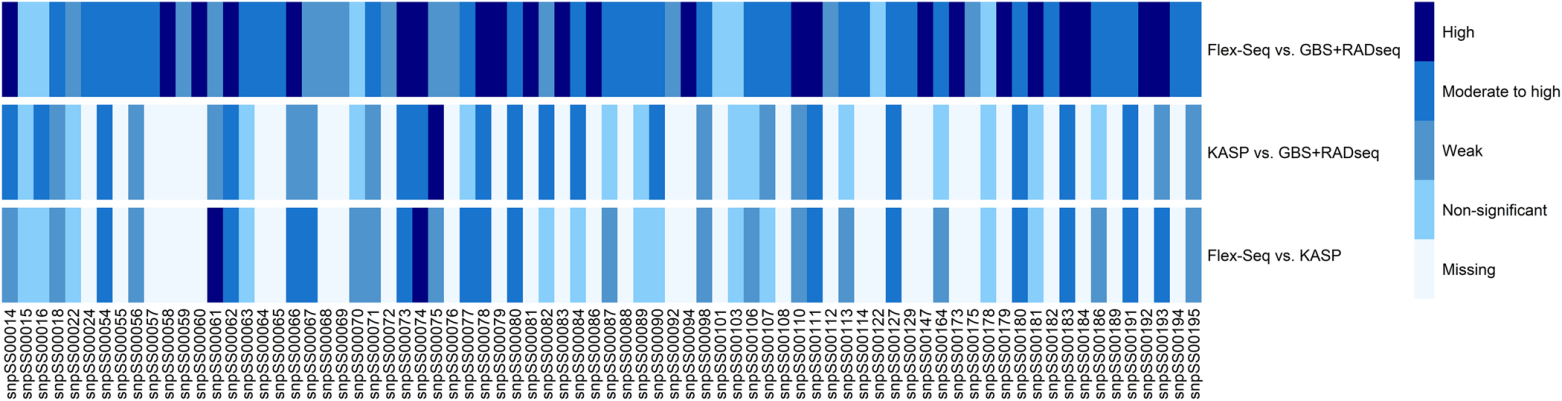
Heatmap of the Pearson product‐moment correlation coefficients for each of the 76 SNP markers selected for sugarcane (*Saccharum* spp.). The categories correspond to high correlation (*r* > |0.80|), moderate‐to‐high correlation (|0.50| < *r*  ≤ |0.80|), weak correlation (|0.23| < *r* ≤ |0.50|), and non‐significant correlation (*r* ≤ |0.23|). The category “missing” corresponds to the markers that could not be converted to KASP.

The allele dosages estimated with Flex‐Seq and GBS+RADseq showed a mean correlation coefficient of 0.62 ± 0.27 (Appendix S3). Although all 76 SNP markers were successfully converted from GBS+RADseq to Flex‐Seq, eight had a non‐significant Pearson correlation for the allele dosage between both technologies with values spanning between −0.23 and 0.23 (Appendix S3). Eleven markers showed a weak correlation (|0.23| < *r* ≤ |0.50|), 33 a moderate‐to‐high correlation (|0.50| < *r* ≤ |0.80|), and the remaining had a high correlation (*r* > |0.80|) (Figure 2), suggesting that Flex‐Seq can reproduce the allele dosages fairly well for more than 75% of informative SNPs in sugarcane.

The fluorescence ratios obtained with KASP technology allowed the estimation of allele dosages for only 42 of the 76 markers (Appendix S3); for the remaining 34 SNPs, the fluorescence ratios did not cluster within the genotypic assignment of a decaploid organism such as sugarcane. When comparing the allele dosages estimated using GBS+RADseq with those obtained with KASP technology for the 42 SNP markers, an average correlation of 0.38 ± 0.27 was observed (Appendix S3). For 14 of the 42 SNP markers, a non‐significant correlation coefficient was observed (Appendix S3), suggesting that, for these markers, the allele dosage estimated with KASP was not able to reproduce the baseline with correlations not significantly different from zero. For the remaining 28 markers, 13 had a weak correlation, 14 had a moderate‐to‐high correlation, and one had a high correlation (Figure 2). Finally, the prediction ability of KASP compared with Flex‐Seq had an average correlation coefficient of 0.38 ± 0.30, with 13 having a non‐significant correlation coefficient (Appendix S3). Within this comparison, there were 14 markers with a weak correlation, 13 with a moderate‐to‐high correlation, and only two with a high correlation (Figure 2).

## DISCUSSION

Both GBS and RADseq are cost‐effective sequencing techniques used primarily to discover large numbers of SNPs, with a broad application potential across distinct species (Cronn et al., 2012). These techniques depend on the discriminatory power of restriction endonucleases, which limits their capability to target specific chromosomal regions or SNPs within the genome (Cronn et al., 2012; Sonah et al., 2022); therefore, an in‐depth analysis is needed to convert the markers discovered with both technologies into another SNP assay format for validation purposes (Cronn et al., 2012). Flex‐Seq offers an alternative approach for marker validation because of its use of highly specific probes to target SNP positions in complex genomes, such as sugarcane, in a reproducible manner (Cronn et al., 2012). Our results showed that Flex‐Seq reproduced the allele dosages of GBS+RADseq with good levels of precision, processing in parallel thousands to millions of reactions per sample of DNA (multiplexing). For Flex‐Seq, the high depth of sequencing (≥200×; the number of times a nucleotide is measured or sequenced in the genome [Cronn et al., 2012; Sims et al., 2014; Jiang et al., 2019; Rapid Genomics, 2023]) significantly decreases the rate of false positives and increases the ability to detect novel variants compared with the other techniques (Jiang et al., 2019).

The biggest challenge in determining the allele dosage of allopolyploid organisms, the genomes of which comprise several subgenomes derived from different wild species (Piperidis and D'Hont, 2020; Aitken, 2022), is the presence of homologous allelic versions with some structural variations. This increases the probability of an erroneous determination of the genotype due to the preferential union between the primers or probes of one allele relative to the other (Makhoul et al., 2020). This phenomenon occurs more frequently when using techniques involving selective amplification with specific primers. KASP, a technique based on uniplex amplifications per sample (He et al., 2014; Ertiro et al., 2015), was less able to differentiate between the variants of the homologous allelic versions, negatively impacting its precision in reproducing the allele dosages of the SNP (Figure 2). It was therefore not possible to reproduce the allele dosages of the genotypes analyzed with the baseline technique (i.e., GBS+RADseq) for most of the SNPs when the scoring and posterior decaploid assignment was carried out using KASP. Indeed, it was not possible to convert 34 of them into KASP. Nevertheless, the allele dosage from a set of markers was reproduced, with 14 of the 42 converted SNPs having a moderate‐to‐high correlation (|0.50| < *r* ≤ |0.80|) and only one displaying a high correlation (i.e., snpSS00075). In polyploid genomes such as wheat (*Triticum aestivum* L.) and sugarcane, Makhoul et al. (2020) and McNeil et al. (2017) found low conversion rates of 20% and 25%, respectively, when using the commercial KASP‐by‐Design services with the flanking sequence regions. They concluded that it is challenging to convert from different marker systems to KASP. By contrast, Gao et al. (2022) found that, of the 77 quantitative trait loci they identified for sugarcane smut resistance, 63% were successfully genotyped in the parents from a biparental population. With low‐complexity genomes such as rice (*Oryza sativa* L.), a conversion rate of 90% (327/364 SNPs) was observed (Steele et al., 2021). Finally, a weaker KASP performance is common when analyzing large and complex genomes such as sugarcane because of the difficulty the technique has in detecting the chromosomal segments that contain the SNP marker of interest (McNeil et al., 2017; Makhoul et al., 2020). Finally, the evaluation of the Pearson correlation coefficients indicated that Flex‐Seq is a viable technique for reproducing the allele dosages from GBS and RADseq in a precise manner for complex and large genomes such as sugarcane, while KASP demonstrated good reproducibility but only for a small set of markers.

## CONCLUSIONS

Flex‐Seq can be used in a high‐throughput genotyping pipeline to target specific positions in the genome by using the parallel sequencing technology of Illumina to evaluate multiple batched samples, reducing the time and cost of processing when validating a SNP marker. By contrast, KASP demonstrated a moderate‐to‐high reproducibility for 14 of 76 markers, and only one marker with a high correlation (*r* > |0.80|), limiting the possible applications of this technique. This suggests that an in‐depth analysis for KASP is required to reproduce the genotype assignment of a decaploid organism. Flex‐Seq is therefore the best‐performing technique to estimate allele dosages for SNP molecular markers in sugarcane in Cenicaña, because of its high precision, ease of implementation, and high‐throughput capability.

## AUTHOR CONTRIBUTIONS

J.J.R. conceived the idea for the evaluation of the sequencing methods that was further analyzed by all authors (H.J., A.L., C.S.‐D., J.H.T.‐M., J.L.‐G., and F.S.A). H.J., A.L., and C.S.‐D. performed the DNA extractions and primer and probe designs where applicable. J.H.T.‐M. completed all bioinformatic analyses of the GBS, RADseq, and Flex‐Seq Ex‐L data. F.S.A. and H.J. analyzed all the KASP and Flex‐Seq data. A.L., C.S.‐D., H.J., and F.S.A. drafted the initial version of the manuscript. All authors contributed to the preparation of the manuscript and approved the final version.

## Supporting information


**Appendix S1**. Composition of Cenicaña's diverse panel of 220 genotypes, representing the genetic diversity of Cenicaña's germplasm bank, wild species, resistant or susceptible to different pests and diseases in Colombia, and genotypes of relevance for the breeding program.

**Appendix S2**. Adjacent window of the 400‐bp (200 bp upstream and 200 bp downstream) sequence used for probe design in the Flex‐Seq and KASP protocols to test the 76 selected SNPs. The SNP is located at position 201 and denoted within a square bracket.

**Appendix S3**. Pearson correlation coefficients between the GBS+RADseq, Flex‐Seq Ex‐L, and KASP results for 53 genotypes of sugarcane (*Saccharum* spp.) and 76 SNP markers. The analysis includes the correlation coefficient, the *t*‐statistic (t), the degrees of freedom (df), and the *P* value (P) for each marker. Empty cells indicate that the corresponding marker was not converted to KASP.

## Data Availability

The scripts and data used in this study are available on GitHub (https://github.com/fsilvaag/Allele_Dosage_Sugarcane_APPS.git). Similarly, the genotypic data are available from the National Center for Biotechnology Information (NCBI) under the accession number PRJNA1040257 (https://www.ncbi.nlm.nih.gov/bioproject/PRJNA1040257).
